# Artificially long legs directly enhance long sprint running performance

**DOI:** 10.1098/rsos.220397

**Published:** 2022-08-17

**Authors:** Peter G. Weyand, Lance C. Brooks, Sunil Prajapati, Emily L. McClelland, S. K. Hatcher, Quinn M. Callier, Matthew W. Bundle

**Affiliations:** ^1^ Locomotor Performance Laboratory, Department of Applied Physiology and Wellness, Southern Methodist University, Dallas, TX, USA; ^2^ Biomechanics Laboratory, School of Integrative Physiology and Athletic Training, University of Montana, Missoula, MT, USA

**Keywords:** prosthetic limb, sprint running, human performance

## Abstract

This comment addresses the incomplete presentation and incorrect conclusion offered in the recent manuscript of Beck *et al*. (*R. Soc. Open Sci.* **9**, 211799 (doi:10.1098/rsos.211799)). The manuscript introduces biomechanical and performance data on the fastest-ever, bilateral amputee 400 m runner. Using an advantage standard of not faster than the fastest non-amputee runner ever (i.e. performance superior to that of the intact-limb world record-holder), the Beck *et al*. manuscript concludes that sprint running performance on bilateral, lower-limb prostheses is not unequivocally advantageous compared to the biological limb condition. The manuscript acknowledges the long-standing support of the authors for the numerous eligibility applications of the bilateral-amputee athlete. However, it does not acknowledge that the athlete's anatomically disproportionate prosthetic limb lengths (+15 cm versus the World Para Athletics maximum) are *ineligible* in both Olympic and Paralympic track competition due to their performance-enhancing properties. Also not acknowledged are the slower sprint performances of the bilateral-amputee athlete on limbs of shorter length that directly refute their manuscript's primary conclusion. Our contribution here provides essential background information and data not included in the Beck *et al.* manuscript that make the correct empirical conclusion clear: artificially long legs artificially enhance long sprint running performance.

## 1. Introduction and background

We appreciate the recent publication of Beck *et al.* and the informative data offered on the sprint running mechanics of this bilateral, lower limb amputee 400 m runner [1]. We also appreciate these authors acknowledging the direct support they have provided to this athlete's eligibility applications and legal proceedings of the last several years. Given our direct involvement in hosting and testing both bilateral-amputee 400 m runners whose data appear in the manuscript [2–4], and our involvement in the eligibility considerations of both [2,5,6], we welcome having biomechanical data from a second Olympic-qualifying, bilateral-amputee 400 m runner available in the literature.

In the case of the Beck *et al.* study, or any objective analysis, reaching valid conclusions requires including the relevant data. Unfortunately, their manuscript does not include either the essential factual background or the data most relevant to the primary scientific question addressed in their manuscript: whether prosthetic legs provide an advantage over biological legs. The manuscript does not acknowledge the athlete who recorded the fastest-ever amputee 400 m time of 44.38 s did so with lower limbs that are ineligible in World Para Athletics competition due to their performance-enhancing properties. The athlete's best performances and all the data presented in Beck *et al*. were achieved on artificial limbs that exceed the maximum allowable Para Athletics lengths by +15 cm [7]. However, the athlete's substantially slower treadmill and race performances on limbs of shorter length [2] are not mentioned.

Notably absent from the Beck *et al*. manuscript is the scientific background responsible for the current views of the scientific, athletic and legal professions on the leg length–performance relationship. As of July 2021, the positive relationship between leg length and bilateral-amputee sprint running performance has been formally recognized by (i) two international sporting organizations [2,7], (ii) leading scientific researchers from three continents whose expertise led to the current Para Athletics limb length policies [8,9] and (iii) the Court of Arbitration for Sport (CAS) panels that considered the issue on behalf of this athlete on two separate occasions [6,10]. The relationship is also acknowledged by Paralympic sprinters themselves, including both the bilateral, lower limb amputee athletes whose data appear in the manuscript [11–13].

## 2. The leg length–sprint performance relationship

### 2.1. Racing leg lengths

Our work with the first Olympic-qualifying, bilateral-amputee 400 m runner over a decade ago led us to then identify two mechanisms by which artificial limbs could be advantageous: (i) reduced limb re-positioning times due to relatively lightweight prostheses and (ii) lengthened steps due to disproportionately long prostheses [4].

**Figure 1.**
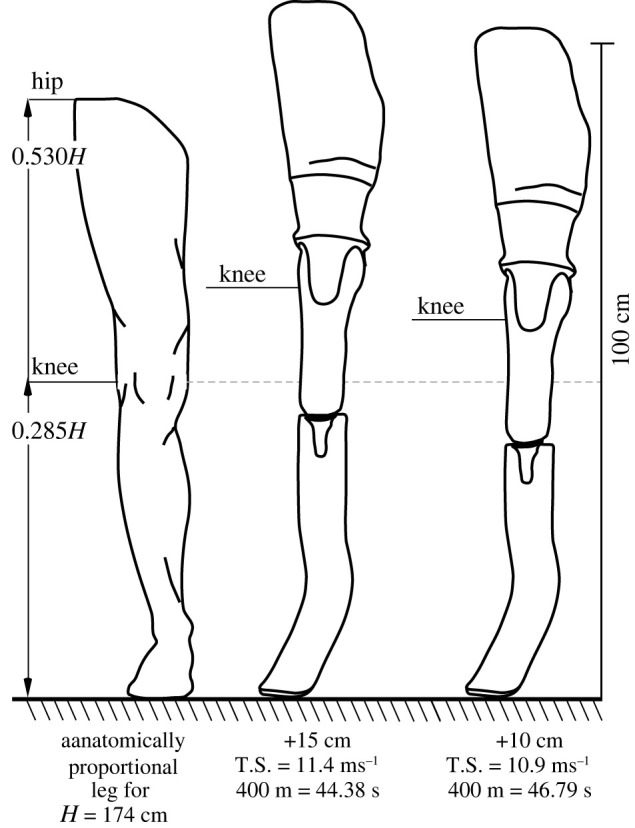
The illustration at left indicates the typical lower limb proportions [16] of an individual of height (*H*) 174 cm (100 cm scale provided at right for reference). Middle (+15 cm) and right (+10 cm) limb plus socket configurations indicate the considerable increase in knee to ground length provided by the respective prostheses. The 5 cm change in prosthetic length (middle versus right illustrations) reduced top treadmill sprint speed by 0.5 m s^−1^ and increased 400 m race times by more than 2 s. Top speeds (T.S.) and mean 400 m race times on the different length lower limbs appear beneath.

In the second case, accurate identification of anatomically disproportionate lower limb lengths for double-limb amputees is straightforward due to the expansive databases that quantify human bodily dimensions, including stature [14–16]. The expansive databases have led to standardized, widely accepted, textbook representations of human body segment proportions like those appearing in figure 1. The most robust of these is used to impose limits on the prostheses lengths of double, lower limb amputees by assigning a maximum allowable standing height (MASH). The MASH formula determines this stature limit for double, lower limb amputees by incorporating sitting height and three intact-limb segment lengths [16]. The current formula was implemented by Para Athletics in 2018, has been both internally and externally [9] validated and has an individual estimation error of 1.91 cm.

The Beck *et al*. manuscript does not report that the 400 m athlete's stature projection with anatomically proportioned lower limbs is 172.5 cm [6,16] with a Para Athletics racing height of 174.4 cm (172.5 + 1.9 cm [7]), nor that his manuscript testing and pre-2021 stature of 189 cm is +15 cm taller than this due to the disproportionate length of his racing prosthetics [6,7,16]. With anatomically proportioned limbs, a +15 cm difference in the below-knee segment would be expected between individuals whose stature varies by 50 cm [14,15].

While the manuscript of Beck and colleagues does not acknowledge the +15 cm difference between the athlete's racing-limb height versus projected height, they have asserted elsewhere [2,10] that the eightfold difference between this athlete's height deviation and the formula's predictive error (15.0 versus 1.91 cm) is attributable to racial bias. However, the race-related differences in segment and torso lengths in question are small (less than 3.0 cm, NHANESIII [17]), offsetting and structured into the MASH formula. Thus, the formula's structure, external validation on subjects of different races [9] and existing anthropometric databases indicate that the racial bias asserted [2,10] vastly exceeds any error margin for which quantitative justification exists.

### 2.2. Sprint performances on different leg lengths

Early in 2021, the athlete began using limbs that were 5 cm shorter than those used prior; on these slightly shortened limbs, he exceeded his MASH height by +10 cm. In 2021, he sprinted on our laboratory treadmill and in track competition on these limbs.

**Figure 2.**
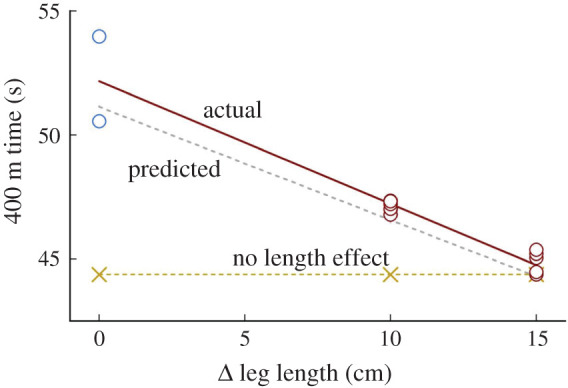
Race times for the 400 m event were slower with shorter prosthetic limbs across *three* leg length conditions for the double lower limb amputee sprinter in Beck *et al*. Differing lengths (i.e. Δ leg length) are referenced to the maximum permitted by the Paralympic formula (i.e. MASH, Δ = 0 cm). The ‘actual’ line (solid) is the line of best-fit to the race time data. The ‘predicted’ line (grey) provides our *a priori* [2] time predictions for the shorter leg length conditions of +10 and 0 cm. (Note: blue circles are 2022 races).

Direct testing in March of 2021 resulted in the outcome we predicted and the CAS recognized in 2020 [6]; the athlete's top treadmill sprinting speed was reduced to 10.9 ms^−1^ [2] versus the 11.4 ms^−1^ on the longer limbs reported in Beck *et al.*'s manuscript [1]. The reduction of 0.5 ms^−1^ in his top speed allowed us to project an increase in his 400 m race times of just over 2 s [18]. Thus, prior race times in the mid-44 s range from the 2018 and 2019 seasons achieved on the +15 cm limbs projected 400 m race time of 46.5 s on the slightly shortened limbs. In six races in April and May of 2021, while attempting to meet the US Olympic Trials qualifying standard, the athlete's best time was 46.79 s and his mean performance time was 47.12 ± 0.22 s (figure 2; electronic supplementary material, table S1). The agreement between the prediction from an early March 2021 treadmill test and track performances in April and May indicates that his fitness level was virtually constant throughout this three-month time frame. These results provide unequivocal evidence that the athlete's performances were substantially enhanced by his disproportionately long legs.

Prior to the 2022 racing season, the athlete further reduced his racing-limb lengths by an additional 10 cm to bring his height into compliance with the MASH formula and Para Athletics length policies. In two subsequent races on MASH compliant limbs, the athlete's best 400 m time was 6.2 s slower versus his personal record achieved on limbs 15 cm longer (50.56 s versus 44.38 s, electronic supplementary material, table S1) in good agreement with our *a priori* prediction of +6.8 s [2].

In fairness to Beck *et al.*, we importantly note that the race data from the spring of 2022 on MASH compliant limb lengths became available after the publication of their manuscript. However, the laboratory and race data on +10 cm limbs were available five months before their manuscript submission.

### 2.3. Racing leg lengths in non-amputee track athletes

**Figure 3.**
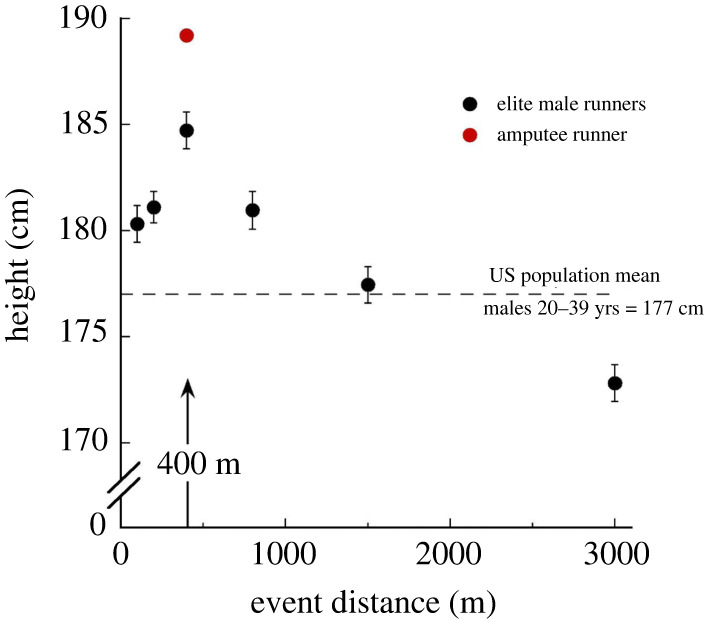
Heights (mean ± s.e.) of the fastest 45 male performers in the world between 1991 and 2003 for track racing events of different distances [19]. The mean stature of the world's fastest 400 m runners exceeds that of the specialists at all other distances, both shorter and longer (identical trend in females; data not shown [19]). The considerably greater height of the bilateral-amputee athlete is entirely attributable to disproportionately lower limbs and results in markedly longer leg lengths than those of the intact-limb competitors.

The well-documented body size optimums for non-amputees in long sprint races provide parallel, independent evidence of the positive relationship between leg length and long sprint performances considered here [19]. Long strides that enable steady-speed, fatigue-resistant sprinting [20–22] are significantly advantageous for the 400 m event while acceleration abilities are relatively unimportant. Consequently, world-class 400 m specialists of both sexes are, on average, substantially taller than specialists at shorter and longer racing distances (figure 3, [19]).

## 3. Concluding remarks

The data of Beck *et al.* [1] appear to have been collected carefully, and we have no reason to consider them invalid. However, the analysis they present is at least as problematic as the essential content they do not include. Here, we note simply that their standard of not ‘*better than that observed by the best non-amputee athlete’* would warrant a ‘no advantage’ conclusion for any amputee athlete who fails to set the World Record.

Given this obviously invalid standard for advantage, omission of essential facts and exclusion of the most relevant data, the Beck *et al*. [1] manuscript offers a conclusion directly refuted by the empirical record. The available data plainly document the artificial enhancement of long sprint running performance made possible by artificially long legs.

## Data Availability

The data used in these analyses are publicly available at the citations provided.
